# Inhibition of allergen‐dependent IgE activity by antibodies of the same specificity but different class

**DOI:** 10.1111/all.12607

**Published:** 2015-03-28

**Authors:** T. S. Dodev, H. Bowen, M. H. Shamji, H. J. Bax, A. J. Beavil, J. M. McDonnell, S. R. Durham, B. J. Sutton, H. J. Gould, L. K. James

**Affiliations:** ^1^ Randall Division of Cell and Molecular BiophysicsKing's College LondonLondonUK; ^2^ MRC and Asthma UK Centre for Allergic Mechanisms of AsthmaKing's College LondonLondonUK; ^3^ Allergy and Clinical ImmunologyNational Heart and Lung InstituteImperial College LondonLondonUK

**Keywords:** allergen, antibody, immunotherapy, isotype, specificity

## Abstract

IgG_4_ purified from patients undergoing specific allergen immunotherapy inhibits the activities of the serum IgE in *in vitro* assays and is thought to reduce the symptoms of the disease. However, it is not known whether this is related to an intrinsic property of this subclass or only the allergen specificity. We tested the hypothesis that allergen specificity is the critical determinant for this activity using a panel of antibodies with identical specificity but different subclasses. The different antibodies were all able to inhibit the activity of IgE to the same extent. We demonstrate that specificity is the dominant factor determining the ability of an antibody to block allergen‐dependent IgE activity.

**Abbreviations**
BAT: basophil activation test
BSA: bovine serum albumin
EDTA: ethylenediaminetetraacetic acid
ELISA: enzyme‐linked immunosorbent assay
FAP: facilitated antigen presentation
HBS: Hepes‐buffered saline
HPLC: high‐performance liquid chromotography
PBS: phosphate‐buffered saline
PIPE: polymerase incomplete primer extension
SCIT: subcutaneous immunotherapy
SPR: surface plasmon resonance
TMB: tetramethybenzidine

Allergy is associated with the excessive production of allergen‐specific IgE. However, allergen‐specific antibodies of other isotypes are produced both in allergic disease and in states of tolerance, for example in hyperimmune beekeepers and in patients treated by allergen immunotherapy 1, 2. The four human IgG subclasses, IgG_1_, IgG_2_, IgG_3_ and IgG_4_, differ mainly in the length and rigidity of the hinge region. These differences impart different functional roles, based on their ability to activate the immune system. Similarly, IgA is represented by two subclasses: IgA_1_, which predominates within the serum, and IgA_2_, with a shorter hinge region and more compact structure, which predominates at mucosal surfaces 3 (Fig. 1A).

**Figure 1:**
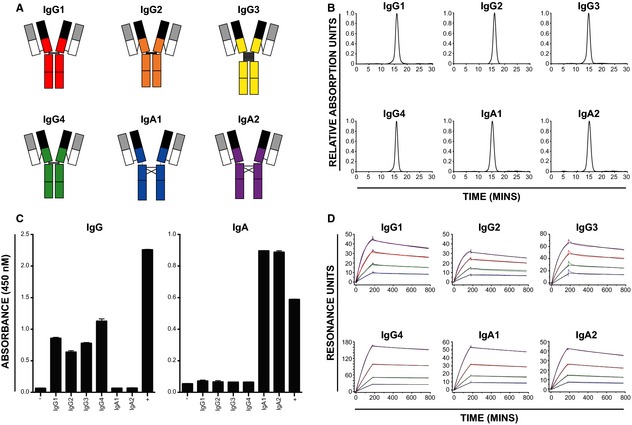
(A) Immunoglobulin domain structures of human IgG_1‐4_ and IgA_1‐2_. Schematic representations of the polypeptide and domain structures of human IgG_1_, IgG_2_, IgG_3_, IgG_4_, IgA_1_ and IgA_2_ showing the intrachain disulphide bridges 3, 13. Variable domains are shown in black and grey. (B) Size exclusion purification profiles. Constant domain‐exchanged Phl p 7‐specific monoclonal antibodies were purified by size exclusion chromatography (Superdex^™^ 200 10/300, flow rate 0.75 ml/min in PBS, pH 7.0). (C) Isotype exchange retains the antibody specificity. ELISA plates were coated with Phl p 7, and antibody binding was confirmed using isotype‐specific detection antibodies. Assay buffer and mixed patient serum were included as negative (−) and positive (+) controls as indicated. (D) Binding kinetics and affinity for Phl p 7 are comparable between antibody isotypes. Recombinant Phl p 7‐specific antibodies were captured on a CM5 sensor chip with a covalently immobilized antilambda antibody. Binding experiments were carried out with twofold serial dilutions of Phl p 7 from a starting concentration of 10 nm. Curves were fit (black lines) to derive on‐ and off‐rates.

Serum from patients receiving allergen immunotherapy blocks the activity of IgE, and this inhibitory activity co‐elutes with the IgG_4_ fraction 4. Furthermore, depletion of IgG_4_ from this serum correlates with a decrease in IgE inhibitory activity 5. However, it is unknown whether blocking activity is restricted to the IgG_4_ subclasses or whether other subclasses that recognize the same epitope are equally effective in blocking IgE‐mediated activity. To test this, we generated a set of recombinant monoclonal antibodies of the same specificity for the grass pollen allergen Phl p 7, with different constant region domains, representing all of the IgG and IgA subclasses. We then measured the affinity of antigen binding and ability of these antibodies to inhibit IgE‐mediated activities in *in vitro* assays.

## Methods

Detailed methods are available in the supporting information.

### Antibody cloning and expression

Matched heavy‐ and light‐chain variable antibody sequences specific to Phl p 7 allergen were previously isolated from a single B cell derived from a patient undergoing grass pollen immunotherapy 6. These sequences were subcloned into the dual antibody expression vector pVITRO1‐102.1F10‐IgG4/λ 7. Phl p 7‐specific human IgG_1,_ IgG_2,_ IgG_3_, and IgA_1_ and IgA_2_ expression vectors were subsequently cloned and expressed using the PIPE method 7.

### Characterization of recombinant antibodies

Human 102.1F10 IgG_1_ and IgG_2_, and IgG_3_ and IgG4 were purified by affinity chromatography with a 5‐ml HiTrap Protein‐G HP column (GE Healthcare Life Sciences, Amersham, UK). Human 102.1F10 IgA_1_ and IgA_2_ were purified by affinity chromatography with immobilized SSL7/Agarose (InvivoGen, Toulouse, France). The purified antibodies were analysed by size exclusion chromatography 14, and specificity was confirmed by Phl p 7 allergen ELISA using biotin‐labelled isotype‐specific antibodies. SPR was performed using a Biacore T200 instrument; antibodies were captured using an immobilized antilambda antibody (Life Technologies Ltd., Paisley, UK), and binding of Phl p 7 (kindly provided by Dr. Rebecca Beavil) was measured using a 3‐min association phase followed by 10‐min dissociation.

### IgE‐facilitated allergen binding (FAB) assay

IgE‐facilitated allergen binding to B cells was performed as previously described 5 using serum from a grass pollen‐sensitized donor (12 ISU Phl p 7‐IgE), recombinant Phl p 7 (kindly provided by Dr. Rebecca Beavil) and purified Phl p 7‐specific antibodies (10 μg/ml) 6, postimmunotherapy serum (SCIT) (patient 102) or assay media (RPMI‐1640).

### Basophil activation assay

Basophil (CD3^−^, CD303^−^, CD294^+^) activation (upregulation of CD63) was measured by flow cytometry following incubation of blood from a Phl p 7‐sensitized donor with recombinant Phl p 7 in the additional presence of Phl p 7‐specific antibodies (10 μg/ml), postsubcutaneous immunotherapy serum (SCIT) (patient 102) or control serum (human AB sera, Lonza, Verviers, Belgium).

## Results

### Antibody characterization

Size exclusion chromatography and ELISA confirmed the purified Phl p 7‐specific IgG_1_, IgG_2_, IgG_3_, IgG4, IgA_1_ and IgA_2_ consisted of monodisperse antibodies of the expected size (Fig. 1B) and specificity (Fig. 1C). Changing the constant region had negligible effects on antibody affinities for Phl p 7 (Fig. 1D), with similar K_D_ values obtained for all antibody subclasses tested (Table S1).

### IgE blocking activity

**Figure 2:**
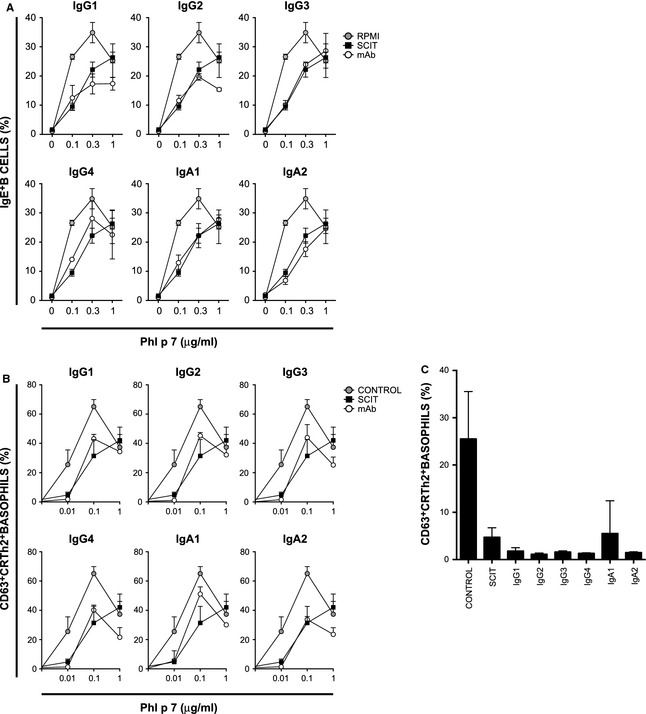
Inhibition of IgE‐facilitated allergen presentation and basophil activation is comparable between different isotypes. Serum containing Phl p 7‐specific IgE was incubated with Phl p 7 in the presence of 10 μg/ml monoclonal antibodies specific to Phl p 7 (open circles). Undiluted immunotherapy serum (SCIT, closed squares) and assay media (RPMI, grey circles) were included as positive and negative controls for IgE blocking, respectively. Binding of IgE‐Phl p 7 complexes was detected by flow cytometry; data are shown as mean ± SEM. B. Basophil activation was detected by flow cytometry following incubation of whole blood from two Phl p 7‐sensitized individuals with increasing concentrations of Phl p 7 and 10 μg/ml monoclonal antibodies specific to Phl p 7 (open circles). Undiluted immunotherapy serum (SCIT, closed squares) and undiluted healthy control serum (control, grey circles) were included as positive and negative controls, respectively. C. Basophil activation following incubation at 10 ng/mL Phl p 7 in the presence of 10 μg/ml monoclonal antibodies specific to Phl p 7. Undiluted immunotherapy (SCIT) serum and healthy control serum were included as positive and negative controls, respectively.

As IgG_4_ has been previously shown to be an effective blocking antibody for IgE‐mediated activity, we wished to determine whether this blocking activity was specific to the IgG_4_ subclass. We therefore tested the IgG and IgA subclasses in two independent *in vitro* assays of IgE activity. Similar to IgG_4_, IgG_1_, IgG_2_, IgG_3_, IgA_1_ and IgA_2_ were able to inhibit binding of IgE‐Phl p 7 complexes to the IgE receptor CD23 (FcεRII) on the surface of B cells (Fig. 2A). In a separate assay, all the monoclonal antibodies tested were able to inhibit IgE‐dependent Phl p 7‐mediated basophil activation to a similar degree (Fig. 2B and 2C).

## Discussion

The inhibitory activity of non‐IgE antibodies in an allergic reaction is thought to be due to their competition with IgE, by masking the epitopes on the allergen. These so‐called blocking antibodies represent a potentially valuable but as yet untested therapeutic commodity for use in passive allergen immunotherapy 8. We previously isolated and cloned a monoclonal IgG_4_ antibody, specific to the grass pollen allergen Phl p 7, from a single B cell isolated from the peripheral blood of a patient treated by specific allergen immunotherapy 6. This single antibody was able to inhibit Phl p 7‐induced IgE activity by up to 60%. This was comparable to the blocking activity of the polyclonal postimmunotherapy serum from the same patient. To test whether this blocking activity was related solely to the specificity or to the (IgG_4_) subclass of the antibody, we compared the ability of other subclasses to inhibit IgE. Although the affinities for Phl p 7 were similar in all of the recombinant antibodies we generated, subtle but significant differences in binding rates were observed (about threefold differences in both on‐ and off‐rate constants, in the most extreme cases), which tended to cancel out to give very similar overall affinities (ranging from 250–570 pm). These differences may reflect subtle conformational changes that constant region domains impart on the variable region, which have been reported to influence the fine specificity and affinity of isotype‐swapped antibodies 9. Nevertheless, we found that the specificity for Phl p 7 was retained and, importantly, isotype exchange had no effect on IgE blocking activity; all subclasses were able to inhibit IgE to nearly the same degree in our *in vitro* assays. Of course, we cannot exclude the possibility that more subtle effects might be observed by titration of the different antibodies. However, under the conditions used here, blocking activities were dependent only on the ability to bind allergen and not on the constant region effector function.

It is almost certain that the blocking activity of an antibody is dependent on several factors such as epitope specificity, concentration and affinity for antigen 10. The affinity of IgE for allergen is an important determinant of its effector function 11, and a blocking antibody must be of approximately equal or higher affinity to prevent IgE binding. Antibody affinity will therefore be a critical factor for selecting blocking antibodies for passive immunotherapy. It is well established that IgG_4_‐expressing B cells secrete the most efficacious blocking antibodies *in vivo* after specific allergen immunotherapy 2. It follows that they would be the best source of heavy‐ and light‐chain genes from which to derive recombinant blocking antibodies for passive immunotherapy. Indeed, IgG_4_ has properties that may favour its use over other IgG subclasses, such as its inability to bind complement, and also the unique property of exchanging one heavy‐/light‐chain pair with an IgG_4_ antibody of a different specificity to generate a bispecific antibody unable to form immune complexes 12.

In summary, our results demonstrate that all IgG and IgA subclasses are capable of inhibiting the activity of IgE in an allergen‐specific manner. Further experiments will be required to examine whether antibodies with the same specificity but different isotype are similarly inhibitory *in vivo* or whether other mechanisms as mentioned above come into play. Phl p 7 is a relatively small allergen, and while our study provides a proof‐of‐concept for passive immunotherapy, it is likely that combinations of allergen‐specific monoclonal antibodies directed against multiple allergens, and possibly multiple epitopes, will be required to ameliorate symptoms depending on the sensitization pattern of each individual 8.

## Author contributions

TSD, HB and HJB produced and characterized antibodies; MHS performed functional assays; JMM helped to design experiments and analyse data; LKJ, AJB, SRD and BJS helped to design experiments and write the manuscript; and LKJ performed and coordinated experiments. HJG proposed the study and helped to write the manuscript.

## Conflict of interest statements

None of the authors have any conflict of interest to declare.

## Supporting information


**Figure S1.** ELISA plates were coated with Phl p 7 and antibody binding was confirmed using subclass‐specific monoclonal detection antibodies.

**Appendix S1.** Methods.
**Table SI.** SPR analysis of the interaction between Phl p 7 and recombinant antibodies (±SE).
