# Supplementary material for: Inhibition of allergen‐dependent IgE activity by antibodies of the same specificity but different class
Source: Allergy. 2015 Mar 28;70(6):720–4. doi: 10.1111/all.12607 (PMC4949685; doi:10.1111/all.12607)
Supplement: Supplementary file 2 — Appendix S1. Methods. Table SI. SPR analysis of the interaction between Phl p 7 and recombinant antibodies (±SE). [file ALL-70-720-s002.docx]

**Supplementary Material**

**METHODS**

**Isotype swapping by PIPE cloning**

A Phl p 7-specific IgG_4_ antibody (6), sub-cloned into the dual antibody expression vector pVITRO1-102.1F10-IgG4/λ (7) was linearised by primer pair pAn_Fwd1 (CTAGCTGGCCAGACATGATAAGATACATTGATGAGTTTGG) and 102.1F10-VH_Rev (TGAGGAGACGGTGACCAGGGCTCCCTGGCCCCAGGAGTCA), flanking the Cγ_4_ region, followed by *Dpn I* (New England Biolabs) treatment. Human antibody constant genes Cγ_1_, Cγ_2_ and Cγ_3_ (Life Technologies Ltd.), were amplified using primer pair 102-Cg1-3_Fwd (CCCTGGTCACCGTCTCCTCAGCTAGCACCAAGGGCCCATC) and Cg1-3_Rev (TATCATGTCTGGCCAGCTAGTCATTTACCCGGAGACAGGG), whereas Cα_1_ and Cα_2_ (Life Technologies Ltd.), were amplified using primer pair 102-Ca1-2_Fwd (CCCTGGTCACCGTCTCCTCAGCTAGCCCGACCAGCCCCAAG) and Ca1-2_Rev (TATCATGTCTGGCCAGCTAGTCAGTAGCAGGTGCCGTCCA). The unpurified linearised pVITRO1-102.1F10-IgG4/λ vector was mixed in 1:1 (v/v) ratio with vector-end homologous PCR amplified Cγ_1_, Cγ_2_, Cγ_3_, Cα_1_ and Cα_2_ and transformed into competent *E. coli* cells (NEB 10-beta) (7), generating Phl p 7 specific human IgG_1,_ IgG_2,_ IgG_3,_ IgA_1_ and IgA_2_ expression vectors, all available at Addgene (<http://www.addgene.org/Andrew_Beavil>).

**Recombinant Antibody Production**

Suspension serum-free adapted FreeStyle^TM^ 293-F cells (Life Technologies Ltd.) were cultured in FreeStyle^TM^ 293 Expression Medium supplemented with 5000 U/ml penicillin and 100 μg/ml streptomycin (Life Technologies Ltd.) and maintained at 37^o^C and 5% CO_2_ in humidified air. FreeStyle^TM^ 293-F cells were transfected with the dual antibody expression vectors pVITRO1-102.1F10-IgG_1_/λ, pVITRO1-102.1F10-IgG_2_/λ, pVITRO1-102.1F10-IgG_3_/λ pVITRO1-102.1F10-IgG_4_/λ, pVITRO1-102.1F10-IgA_1_/λ and pVITRO1-102.1F10-IgA_2_/λ (7) using FuGENE HD (Promega) and selected in medium containing 50 μg/ml Hygromycin (Life Technologies Ltd.). Stably transfected cells were expanded for 14 days then supernatants were harvested and filtered.

**Purification of Recombinant Antibodies**

Human 102.1F10 IgG_1-4_ were purified by affinity chromatography with a 5 ml HiTrap Protein-G HP column (GE Healthcare Life Sciences) as previously described (7). Human 102.1F10 IgA_1_-_2_ were purified by affinity chromatography with immobilised SSL7 / Agarose (InvivoGen).

**Size exclusion chromatography**

Purified antibodies were analysed by size exclusion chromatography as previously described (14). Briefly, gel filtration was performed on a Gilson HPLC system using a Superdex™ 200 10/300 GL column (GE Healthcare Life Sciences) at a flow rate of 0.75 ml/min in PBS (pH 7.0).

**Grass pollen allergen and isotype-specific ELISA**

Allergen specificity was confirmed by ELISA of cell-free supernatants using ELISA plates (Maxisorp, Nunc) coated with 10 μg/ml recombinant Phl p 7 allergen (kindly provided by Dr. Rebecca Beavil, MRC & Asthma UK Protein Production Facility, King’s College London, UK) and detection using either Peroxidase-conjugated isotype specific polyclonal antibodies against human IgA and IgG (both Sigma) or biotin-labeled subclass specific monoclonal antibodies; IgG_1_ (clone 8c/6-39, Sigma-Aldrich); IgG_2_ (clone HP-6014, Sigma-Aldrich); IgG_3_ (clone HP-6050, Sigma-Aldrich); IgG_4_, incubated at 1 µg/ml, (clone G17-4, BD Biosciences), followed by streptavidin–horseradish peroxidase (R&D Systems). HRP-labeled anti-human antibodies were used to detect IgA_1-2_ and IgE; IgA_1_ (clone B3506B4, Abcam); IgA_2_ (clone A9604D2, Abcam); IgE (Sigma-Aldrich). All antibodies were used at recommended dilutions (unless stated otherwise).

**Surface plasmon resonance (SPR)**

SPR was performed using a Biacore T200 instrument. A CaptureSelect^TM^ Anti-lambda antibody (Life Technologies Ltd.) was immobilized on a CM5 chip (GE Healthcare Life Sciences) by amine coupling. Antibodies of all isotypes were captured using a 5-minute injection, and binding of Phl p 7 (kindly provided by Dr. Rebecca Beavil) was measured using a 3-minute association phase and 10-minute dissociation. Affinity and rate constants were calculated using BIAevalution software, version 3.2 (GE Healthcare Life Sciences)

**IgE-Facilitated Allergen Binding (FAB) assay**

EBV-transformed B cells (1x10^5^ cell per test) were incubated with serum from a grass pollen-sensitised donor (12 ISU Phl p 7-IgE), recombinant Phl p 7 (kindly provided by Dr. Rebecca Beavil) and purified Phl p 7-specific antibodies (10 μg/ml), post-immunotherapy serum (SCIT) (patient 102) or assay media (RPMI-1640) for 1 hour at 4°C. Cells were washed and binding of Phl p 7-IgE complexes was detected using anti-IgE-PE (Miltenyi). Data were acquired using a BD FACSCanto II system (BD Bioscience) and analysed with FACS DIVA software (BD Bioscience).

**Basophil Activation Assay**

100 μl of heparinized blood from a Phl p 7-sensitised donor was incubated with recombinant Phl p 7 in the additional presence of Phl p 7-specific antibodies (10 μg/ml), post-immunotherapy serum (SCIT) (patient 102) or control serum (human AB sera, Lonza). Cells were stained with anti-human CD3 (PE-Cy7), CD303 (APC) and CD294 (CRTH2) (PE) to identify basophils (CD3^-^, CD303^-^, CD294^+^) and activation determined by upregulation of CD63 (FITC). Erythrocytes were lysed (FACS lysing solution, BD Biosciences) prior to acquisition and analysis as described.

**Supplementary Online Results**

**TABLE EI.** SPR analysis of the interaction between Phl p 7 and recombinant antibodies (±SE)

|  | **k_on_ (M^-1^ s^-1^)** | **k_off_ (s^-1^)** | **K_D_ (M)** |
| --- | --- | --- | --- |
| **IgG_1_** | 1.2(±0.2)x10^6^ | 3.5(±0.5)x10^-4^ | 2.9x10^-10^ |
| **IgG_2_** | 1.5(±0.3)x10^6^ | 3.7(±0.6)x10^-4^ | 2.5x10^-10^ |
| **IgG_3_** | 1.1(±0.2)x10^6^ | 2.9(±0.4)x10^-4^ | 2.6x10^-10^ |
| **IgG_4_** | 5.2(±0.5)x10^5^ | 1.4(±0.2)x10^-4^ | 2.7x10^-10^ |
| **IgA_1_** | 6.2(±0.6)x10^5^ | 2.0(±0.2)x10^-4^ | 3.2x10^-10^ |
| **IgA_2_** | 5.4(±0.4)x10^5^ | 3.1(±0.3)x10^-4^ | 5.7x10^-10^ |

**Supplementary Figure 1 Legend**

ELISA plates were coated with Phl p 7 and antibody binding was confirmed using subclass-specific monoclonal detection antibodies. Assay buffer and mixed patient serum were included as negative (-) and positive (+) controls as indicated.
